# A perspective on the *ORS Spine Section* initiative to develop a multi‐species *JOR Spine* histopathology series

**DOI:** 10.1002/jsp2.1165

**Published:** 2021-07-26

**Authors:** Chitra L. Dahia, Julie B. Engiles, Sarah E. Gullbrand, James C. Iatridis, Alon Lai, Christine L. Le Maitre, Jeffrey C. Lotz, Koichi Masuda, Cheryle A. Séguin, Marianna A. Tryfonidou

**Affiliations:** ^1^ Orthopedic Soft Tissue Research Program Hospital for Special Surgery New York City NY USA; ^2^ Department of Cell and Developmental Biology Weill Cornell Medicine, Graduate School of Medical Sciences New York New York USA; ^3^ Department of Pathobiology, New Bolton Center, School of Veterinary Medicine University of Pennsylvania Kennett Square Pennsylvania USA; ^4^ Corporal Michael J. Crescenz VA Medical Center Philadelphia Pennsylvania USA; ^5^ McKay Orthopaedic Research Laboratory, Department of Orthopaedic Surgery University of Pennsylvania Philadelphia Pennsylvania USA; ^6^ Leni and Peter W. May Department of Orthopaedics Icahn School of Medicine at Mt. Sinai New York New York USA; ^7^ Biomolecular Sciences Research Centre Sheffield Hallam University Sheffield UK; ^8^ Department of Orthopaedic Surgery University of California San Francisco California USA; ^9^ Department of Orthopaedic Surgery University of California San Diego California USA; ^10^ Department of Physiology & Pharmacology Schulich School of Medicine & Dentistry, Bone and Joint Institute, The University of Western Ontario London Ontario Canada; ^11^ Department of Clinical Sciences, Faculty of Veterinary Medicine Utrecht University Utrecht The Netherlands

**Keywords:** human, large animal, mouse, rabbit, rat

## Abstract

This perspective summarizes the genesis, development, and potential future directions of the multispecies *JOR Spine* histopathology series.

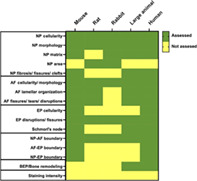

Improving our capability to prevent, diagnose, and treat intervertebral disc degeneration and associated painful conditions requires integration of data from many model systems, including computational simulations, cell and organ culture, small and large animals, as well as human tissue and clinical studies. Maximizing clinical and scientific impact depends upon thoughtful leveraging of observations across systems. Fundamental to success is that results are rigorous, broadly reproducible, generalizable, and ultimately interpretable relative to the human situation. Histopathology is a fundamental and ubiquitous method for evaluating the intervertebral disc and surrounding structures. Yet, to date no commonly accepted histology scoring systems exist in the spine field; in contrast in the cartilage field, the OARSI and ICRS scoring systems are utilized for evaluating cartilage degeneration and repair.^1^, ^2^ In June 2019, the editors of *JOR Spine* in collaboration with the *ORS Spine Section* tasked the community to generate a series of histopathology scoring systems to improve cross‐species comparisons of animal or human features characteristic of disc degeneration and regeneration where relevant and available.^3^ Volunteer leaders reviewed literature, organized conference calls with spine scientists across the globe and developed recommendations for scoring systems specific for mouse, rat, rabbit, large animal models, or human intervertebral discs. After a herculean effort by all involved, this special issue is a result of that call to action. The purpose of this special issue is to share best practices for documenting and reporting histopathologic features of in vivo models for intervertebral disc degeneration and regeneration. Standardization of tissue processing, feature classification, and reporting methods is critical to advance the field. As such, the studies presented here are a valuable contribution to the field of comparative spine pathology, and will also motivate future efforts to share best practices and training materials.

This special issue contains manuscripts outlining guidance for histologically evaluating human disc degeneration,^4^ in addition to commonly used preclinical animal models such as mouse,^5^ rat,^6^ rabbit,^7^ and large animal models,^8^ including dogs, goats, pigs, and sheep. The mouse, rat, and rabbit manuscripts aim to provide comprehensive histopathological scoring systems applicable to multiple models of degeneration and/or repair within each species, with the goal of providing a platform to allow for the more robust comparison of data across studies and research groups. The large animal model manuscript provides a basic toolbox for evaluating degeneration in various models that extends beyond histopathology, incorporating directions for macroscopic, biomechanical, biomolecular, and clinical parameters. This toolbox is meant to be applicable to all large animal models independent of the spinal segment selected and the specific aim of the study. Finally, the manuscript focused on human disc tissues provides a contemporary system for characterizing the features of human disc degeneration that will allow consistent and reproducible linkages to clinical information and imaging to establish relevance and provide a reference standard against which animal data should be evaluated to address applicability to the human situation. Common features shared among all scoring systems are summarized in Figure 1. Approaches for all species included scoring of features within the annulus fibrosus, nucleus pulposus, and endplate. While the human disc histopathological scoring system did not have a separate scoring category for the interface or boundary region, these features were included in scoring the criteria for each region. No scoring system incorporated staining intensity as a feature as this may vary largely depending on the tissue processing and protocols employed. Figure 2A summarizes the maximum scores obtainable for degenerated discs in each system. Figure 2B provides the percentage of the total score driven by each disc component, hence, summarizing the relative weighting of each feature.

**FIGURE 1:**
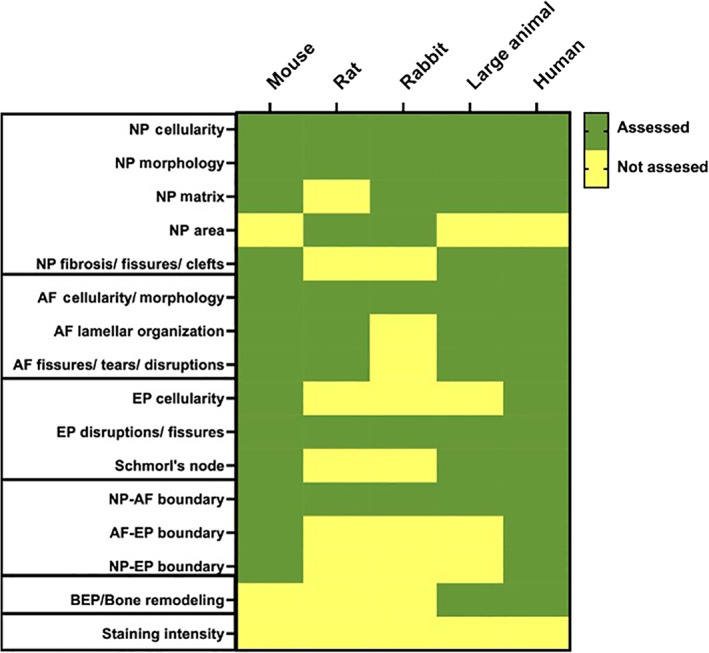
Summary of the features assessed or not assessed in each histopathological scoring system

**FIGURE 2:**
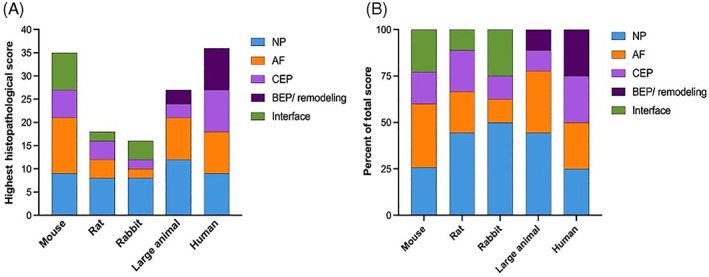
Comparison of the histopathologic score in each scoring system, stratified by scoring category (A), and the relative percentage of each category contributing toward the total score (B)

The histopathology scoring systems described in these manuscripts were primarily constructed via in‐depth survey and/or comparative analysis of the existing scoring systems for each species described in the literature. The groups focused on mouse, rat, rabbit, and human also solicited direct input from the field by sending surveys to *ORS Spine Section* members and authors of recent publications using these species regarding their opinion on which categories would be important to be included in a standardized scoring system. The groups focused on mouse, rat, rabbit, and human then tested and validated their proposed scoring systems and used that information for refinement. The large animal model scoring system has not yet been validated but is based on readily available and well‐validated systems.

The development of these scoring systems was certainly not without its challenges. Each of the groups contemplated issues centered around the heterogeneity within animal species with respect to (subtle) differences in anatomy, varying techniques for inducing degeneration, or the response observed with repair. For example, in mouse models, endplate structure varies with skeletal maturity among mouse strains, so the group needed to narrow down features to include in the scoring system that were always observable, but also changed with pathology. Degeneration studies involving the rat model are frequently performed in two different regions of the spine (lumbar and coccygeal regions) and a wide variety of methods are used to induce degeneration. As such, the group spent time assessing and considering these different rat models of degeneration as well as disc levels, and ultimately decided to use uniform descriptions and categories. While these scores and categories should be relevant across these different models and levels to enable cross‐study comparisons, their manuscript highlights that appropriate controls should be included within studies to best contextualize findings and grading. In the rabbit model, some of the cellular features of degeneration compared to repair are quite distinct and therefore difficult or impossible to capture in a single scoring system. The rabbit group extensively deliberated how to make the scoring system as simple as possible, yet applicable to both degeneration and regeneration models, and ultimately decided to propose a “main” scoring system that could be used for all studies in the rabbit model, with an “addendum” scoring system to be used only for studies of repair/regeneration. The large animal group was challenged by the variability within, but primarily between the four commonly used large animal species predominantly used in a preclinical setting to provide proof‐of‐concept. In large animal models, complementary outcomes studying disc degeneration/regeneration in a single disc are possible but not yet widely used. Therefore, the team focused on bringing first available expertise and experiences to create a comprehensive toolbox for anatomical and functional outcomes. The human group had the unique challenge of incorporating a large range of magnification into their scoring system, as the significance of important features needed to be evaluated over a range of length scales. This was further hampered by the decreased access to microscopes due to the COVID‐19 pandemic to enable the group working on human discs and large animal models to capture whole discs at a quality to enable the viewer to zoom into the area. Thus, “mock” human discs were compiled utilizing images submitted by the spine community to represent whole disc images and high magnification regions representative of features which could be identified in such human and large animal discs to enable testing of the scoring system.

We expect these manuscripts will provide a standardized and useful resource for the field. All papers in this series involved broad considerations and input, and we therefore anticipate these scoring systems will be widely used to facilitate their improvement and advance disc research with better scientific comparisons and reproducibility across different labs. The validation studies performed in several of these manuscripts have clearly highlighted the importance of training graders prior to their use of any histopathology scoring system. To encourage the widespread adoption of these scoring systems by the field, we plan to develop and disseminate training modules, and conduct training workshops at future in‐person and virtual meetings. Such training sessions could inform a larger community on analysis methods for histological scoring of discs. Highlighting these methods and broader usage also helps clarify the limitations of any scoring system.

This series of papers represents a scientific record of the current state; yet no one paper incorporates all ideas, and science always advances. All groups identified future efforts which may be undertaken by the field and presented in a complementary series of work, for example, the validation of regeneration/repair scores in those model species for which such a score has not already been proposed, the role of sex and genetics in animal degeneration models, or developing guidance on other outcome metrics for assessing degeneration (ie, imaging methods, pain/behavioral assays). Knowledge gained from the outcomes of each model can generate robust evidence which enables alignment with features of human disc degeneration and can thereby better apply to the human situation. We believe these papers provide a robust framework for improved comparison across labs and would consider the success of this series to be the stimulation of active discussions, providing a dynamic evolution with scoring system improvements as they are applied in practice so as to improve clinical care.
